# Influence of spacer length on heparin coupling efficiency and fibrinogen adsorption of modified titanium surfaces

**DOI:** 10.1186/1475-925X-6-31

**Published:** 2007-07-17

**Authors:** David Tebbe, Roger Thull, Uwe Gbureck

**Affiliations:** 1 Department for Functional Materials in Medicine and Dentistry, University of Würzburg, Pleicherwall 2, D-97070 Würzburg, Germany

## Abstract

**Background:**

Chemical bonding of the drug onto surfaces by means of spacer molecules is accompanied with a reduction of the biological activity of the drug due to a constricted mobility since normally only short spacer molecule like aminopropyltrimethoxysilane (APMS) are used for drug coupling. This work aimed to study covalent attachment of heparin to titanium(oxide) surfaces by varying the length of the silane coupling agent, which should affect the biological potency of the drug due to a higher mobility with longer spacer chains.

**Methods:**

Covalent attachment of heparin to titanium metal and TiO_2 _powder was carried out using the coupling agents 3-(Trimethoxysilyl)-propylamine (APMS), *N*- [3-(Trimethoxysilyl)propyl]ethylenediamine (Diamino-APMS) and *N*^1^- [3-(Trimethoxy-silyl)-propyl]diethylenetriamine (Triamino-APMS). The amount of bound coupling agent and heparin was quantified photometrically by the ninhydrin reaction and the tolidine-blue test. The biological potency of heparin was determined photometrically by the chromogenic substrate Chromozym TH and fibrinogen adsorption to the modified surfaces was researched using the QCM-D (Quartz Crystal Microbalance with Dissipation Monitoring) technique.

**Results:**

Zeta-potential measurements confirmed the successful coupling reaction; the potential of the unmodified anatase surface (approx. -26 mV) shifted into the positive range (> + 40 mV) after silanisation. Binding of heparin results in a strongly negatively charged surface with zeta-potentials of approx. -39 mV. The retaining biological activity of heparin was highest for the spacer molecule Triamino-APMS. QCM-D measurements showed a lower viscosity for adsorbed fibrinogen films on heparinised surfaces by means of Triamino-APMS.

**Conclusion:**

The remaining activity of heparin was found to be highest for the covalent attachment with Triamino-APMS as coupling agent due to the long chain of this spacer molecule and therefore the highest mobility of the drug. Furthermore, the adsorption of fibrinogen on the differently heparinised surfaces in real time demonstrated that with longer spacer chains the ΔD/Δf ratios became higher, which is also associated with better biocompatible properties of the substrates in contact with a biosystem.

## Background

Cardiovascular diseases determined by arteriosclerosis are the main cause of death in developed nations [1]. Since surgical interventions strongly stress the patient's organism, modern minimal invasive methods have become more and more important [2]. During the last two decades, intravascular coronary stent implantation at the site of acute artery closure has been widely used and has increased the quality of life and life expectancy of patients with coronary diseases. This type of surgery has proved effective in restoring vessel potency and decreasing myocardial ischemia. 316L stainless steel and titanium(alloys) are widely used in the fabrication of coronary stents because of their electrochemical and mechanical properties [3]. However, the exposure to blood flow can result in thrombus formation and smooth muscle cell proliferation, which both ultimately lead to restenosis. To avoid thrombus formation, aggressive anticoagulants are systemically applied, however, this can cause bleeding disorder in long-term application. Thus, a great amount of recent work has attempted to develop non-thrombogenic coatings for these metallic stents, e.g. by chemical coupling of antithrombogenic drugs (heparin, heparan sulfate) to the surface [4,5].

**Figure 1:**
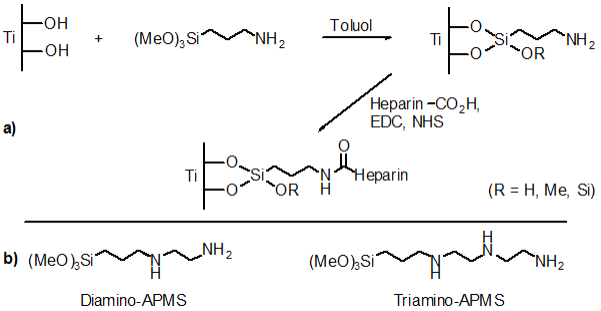
a) Reaction scheme of the immoblilisation of heparin on titania by dint of APMS and b) further spacer molecules used as coupling agents for heparin.

This work aimed to investigate the influence of different coupling agents with varying chain length on the amount and biological performance of surface bound heparin. The principle of the surface modification is demonstrated in Figure 1a for 3-(Trimethoxysilyl)-propylamine (APMS) as commonly used spacer. Alternative coupling agents (Figure 1b) with two or three amino-moieties were *N*- [3-(Trimethoxysilyl)propyl]ethylenediamine (Diamino-APMS) and *N*^1^- [3-(Trimethoxysilyl)-propyl]diethylenetriamine (Triamino-APMS). Surface modification was performed using both TiO_2 _powder and titanium sheets as substrates. The coupling reaction was followed by means of zeta-potential measurements and the amount of surface bound spacer and heparin and their hydrolytic stability was quantified via the ninhydrin and the toluidine-blue reaction. Determination of the biological activity of the immobilised heparin was performed with the chromogenic substrate Chromozym TH test. Fibrinogen adsorption to the modified surfaces was followed in real time using Quartz Crystal Microbalance with Dissipation Monitoring (QCM-D).

## Methods

### Materials

Titanium dioxide (anatase) was purchased from Merck (Darmstadt, Germany). The coupling agents 3-(Trimethoxysilyl)-propylamine, *N*- [3-(Trimethoxysilyl)propyl]ethylenediamine and *N*^1^- [3-(Trimethoxysilyl)-propyl]diethylenetriamine were obtained from Merck (Darmstadt, Germany), Fluka (Buchs, Schwitzerland) and Aldrich (Steinheim, Germany). *N*-(3-Dimethylaminopropyl)-*N*'-ethylcarbodiimide Hydrochloride(EDC) was purchased from Sigma Chemical Company. *N*-Hydroxysuccinimide (NHS) was purchased from Merck. 2-(*N*-Morpholino)ethanesulfonic acid (MES) and Heparin Sodium salt were purchased from Fluka (Buchs, Schwitzerland).

### Silanisation of titanium dioxide and cp-titanium

Modification of TiO_2 _was carried out under variation of a published preparation regime [6]. 2.00 g TiO_2 _(25.0 mmol) and 2.00 ml 3-(Trimethoxysilyl)-propylamine (APMS, 11.3 mmol) were suspended under argon atmosphere in 50.0 ml anhydrous toluene following 2 h treatment with ultrasound (35 kHz, Sonorex RK 102, Fa. Bandelin, Berlin). Afterwards, the functionalised TiO_2 _was separated with a G4-frit from the solution, washed with toluene and then purified with ethyl acetate in a Soxleth-extractor for 2 h. Finally, the sample was dried in vacuum at room temperature. The reaction was alternatively performed with 2.46 ml *N*- [3-(Trimethoxysilyl) propyl]ethylenediamine (Diamino-APMS, 11.3 mmol) or 2.91 ml *N*^1^- [3-(Trimethoxysilyl)-propyl]diethylenetriamine (Triamino-APMS, 11.3 mmol).

cp-Titanium surfaces were modified in a similar manner. The substrates (d = 16 mm, h = 1 mm; Zapp, Düsseldorf) were firstly cleaned in 5% EXTRAN solution for 10 min using ultrasound following three washes with deionised water. Oxidation of the metal surface to produce a homogeneous oxide layer was either performed by using a solution of conc. H_2_SO_4_/30% H_2_O_2 _(1:1) for 3.5 h at room temperature or thermally by annealing the substrates at 750°C for 90 min in a furnace. These samples were then boiled in a solution of 7.00 ml APMS (39.7 mmol) in 70 ml toluene for 6 h.

### Covalent attachment of heparin

In each case 500 mg of the functionalised TiO_2 _was suspended in 50.0 ml MES-buffer (50.0 mM, 40% (v/v) ethyl alcohol/water, pH = 5.5) and stirred for 15 min. Then, 95.0 mg *N*-(3-Dimethylaminopropyl)-*N*'-ethylcarbodiimide Hydrochloride (EDC, 0.50 mmol), 12.0 mg *N*-Hydroxysuccinimide (NHS, 0.10 mmol) and 100 mg heparin (~5.60 μmol) were added to the suspensions followed by stirring the reaction mixture at room temperature for 6 h. Finally, the solid was separated by centrifugation and the obtained modified powders were washed three times each with Na_2_HPO_4_-solution (0.10 M), NaCl-solution (2.00 M), deionised water and then dried in vacuum at 40°C over night. The modification of APMS modified titanium substrates with heparin was performed in a similar manner by stirring the metal samples in a solution of 19.0 mg EDC (0.10 mmol), 2.00 mg NHS (0.02 mmol) and 20.0 mg heparin in 50 ml MES-buffer for 6 h at room temperature.

### Quantification of surface bound spacer and heparin

The quantification of the spacer was determined by the amount of terminal primary amino groups and could be measured photometrically by dint of the ninhydrin reaction after Moore and Stein [7-9]. Basically, this method is based upon the reaction of primary amino groups with ninhydrin to give the dye "Ruhemanns Purpur", which can be quantified photometrically at 570 nm. The amount of immobilised heparin was quantified by means of the toluidine-blue method, already described in literature [10]. This method is based on complexation of the dye toluidine blue with heparin in aqueous medium. By the use of an excess of the dye, the residual "free" toluidine-blue can be determined photometrically at 631 nm and can be correlated with the amount of immobilised heparin.

### Hydrolysis behaviour of immobilised heparin

Five probes with 250 mg of the TiO_2 _powders, respectively, modified with APMS, Di- and Triamino-APMS and heparin were given in glass flasks. Then, 5.00 ml PBS buffer was added and the suspensions were treated with ultrasound for 3 min. The flasks were densely closed with a plastic lid and then given inside an incubator (37°C) onto a shaker (70 shakes per minute). At scheduled times one probe of each heparinised samples was taken, the powder was separated from the liquid with a centrifuge and then dried in vacuum. The amount of residual immobilised heparin was quantified photometrically with the toluidine-blue method.

### Determination of the biological potency of heparin

For the determination of the biological activity of the immobilised heparin, the chromogenic substrate Chromozym TH was used. The principle of this method is based upon heparin formation with antithrombin (AT-III), a heparin/AT-III-complex in an aqueous medium. In the presence of an excess of thrombin, formation of a heparin/AT-III/thrombin-complex follows. Excessive thrombin in solution catalyses the hydrolysis of the chromogenic substrate Chromozym TH into a dye, which can be measured photometrically at 405 nm [11]. The higher the biological activity of the heparin remains after immoblisation, the more thrombin is complexed and the less dye will be hydrolysed.

### Surface charge (Zeta-potential) of modified surfaces

Zeta potentials were measured with a Zeta-sizer 3000 (Malvern Instruments, Herrenberg, Germany) equipped with a HeNe-Laser (λ = 633 nm) and a standard electrophoresis cell at an electrical field force of ± 150 mV. The suspension medium was double distilled water. For data compilation the software PCS version 1.36 from Malvern Instruments, Herrenberg was used.

### Fibrinogen adsorption to modified surfaces

The adsorption of fibrinogen onto the modified surfaces was measured in real-time using Quartz Crystal Microbalance with Dissipation Monitoring (QCM-D; Q-Sense, D300, Sweden). Modification of the quartz crystals was performed in three steps: firstly a thin TiO_2 _coating was applied using physical vapour deposition (PVD) technique [12] following chemical coupling of APMS, Diamino-APMS and Triamino-APMS and heparin similar to the methods described for cp-titanium samples. Fibrinogen adsorption was measured at room temperature using 5.00 ml of a solution of fibrinogen dissolved in phosphate buffered saline (PBS, 50.0 μg fibrinogen/ml PBS) for about 5 h. The adsorbed mass of fibrinogen on the differently heparinised surfaces was calculated according to the Sauerbrey equation, where the change in the resonance frequency Δf is proportional to the change in the adsorbed mass Δm:

Δm=−CnΔf
 MathType@MTEF@5@5@+=feaafiart1ev1aaatCvAUfKttLearuWrP9MDH5MBPbIqV92AaeXatLxBI9gBaebbnrfifHhDYfgasaacH8akY=wiFfYdH8Gipec8Eeeu0xXdbba9frFj0=OqFfea0dXdd9vqai=hGuQ8kuc9pgc9s8qqaq=dirpe0xb9q8qiLsFr0=vr0=vr0dc8meaabaqaciaacaGaaeqabaqabeGadaaakeaacqqHuoarcqWGTbqBcqGH9aqpcqGHsisldaWcaaqaaiabdoeadbqaaiabd6gaUbaacqqHuoarcqWGMbGzaaa@36A7@

with n being the number of the overtone (= 3) of the measured frequency and C = 17.7 ng/cm^2^Hz for the crystals used in this experiment [13].

## Results

**Figure 2:**
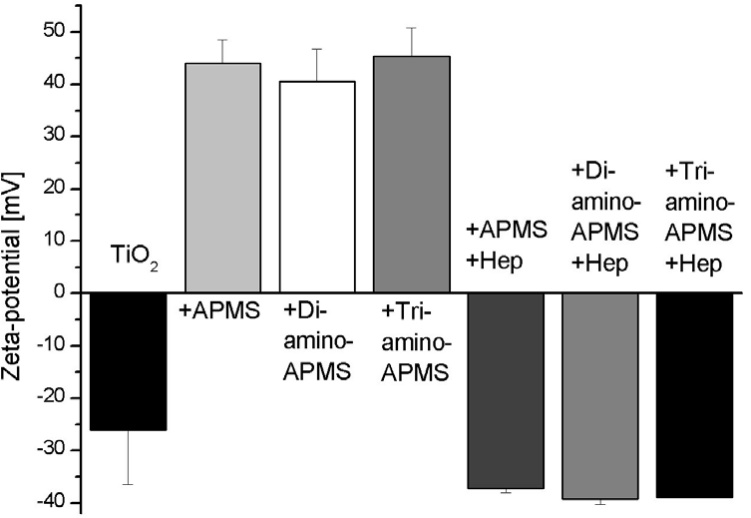
Zeta potentials after functionalisation of TiO_2 _powder with APMS, Di- and Triamino-APMS and respectively after the reaction with heparin. Each data point is the mean of three samples, error bars are standard deviations.

Chemical coupling of silane spacers and heparin to TiO_2 _was followed by measuring the zeta-potentials from the unmodified, with spacer functionalised or rather heparinised powders (Figure 2). Unmodified TiO_2 _had a negative zeta potential of -26.1 ± 10.5 mV due to its deprotonated hydroxyl groups at pH 7 in water. After modification with the different spacer molecules, the zeta-potentials turned positive to + 44.1 ± 4.4 mV (APMS), + 40.6 ± 6.2 mV (Diamino-APMS) and 45.3 ± 5.5 mV (Triamino-APMS), due to protonated primary amino groups (RNH_3 _^+^) of the spacer molecules in water. Finally, the covalent attachment of heparin changes the zeta-potentials to negative values of -37.2 ± 0.9 mV (APMS), -39.3 ± 1.0 mV (Diamino-APMS) and 39.0 ± 0.2 mV (Triamino-APMS). The strong decrease of the zeta-potentials is caused by negative sulphate and carboxyl groups of the drug in the aqueous medium at pH 7.

The amount of covalently attached primary amino groups on the titania surface (powder and metal sheets) was determined photometrically by the ninhydrin reaction and is shown in Table 1. The amount of immobilised terminal amino groups on TiO_2 _powder was the highest for APMS and decreased for Di- and Triamino-APMS.

**Table 1:** Quantification of NH_2 _groups per area for different spacer molecules on cp-titanium and on TiO_2 _powder (amount per powder weight in [nmol/mg] in parenthesis)

Substrate	Number of primary NH_2_-groups per nm^2^		
	
	APMS	Diamino-APMS	Triamino-APMS
cp-Ti disc	156 ± 16	162 ± 12	134 ± 22
TiO_2 _powder	20.0 ± 0.5 (84.9 ± 2.1)	18.7 ± 0.5 (78.7 ± 2.0)	17.0 ± 0.3 (71.3 ± 1.3)

Quantification of immobilised heparin was done by the toluidine-blue method and was only applicable for the drug modified powders (Table 2). On the Ti discs, no heparin was detectable by this method, probably because the amount of bound drug was less than the detection limit of 10 μg by this method. On the APMS spacer, the greatest amount of 53.3 ± 3.6 ng/cm2 heparin was attached compared to Di- and Triamino-APMS (Table 2).

**Table 2:** Quantification of heparin immobilised on APMS, Di- and Triamino-APMS modified titania powders

	APMS	Diamino-APMS	Triamino-APMS
[ng/cm^2^]	53.3 ± 3.6	41.2 ± 6.9	32.1 ± 5.7

**Figure 3:**
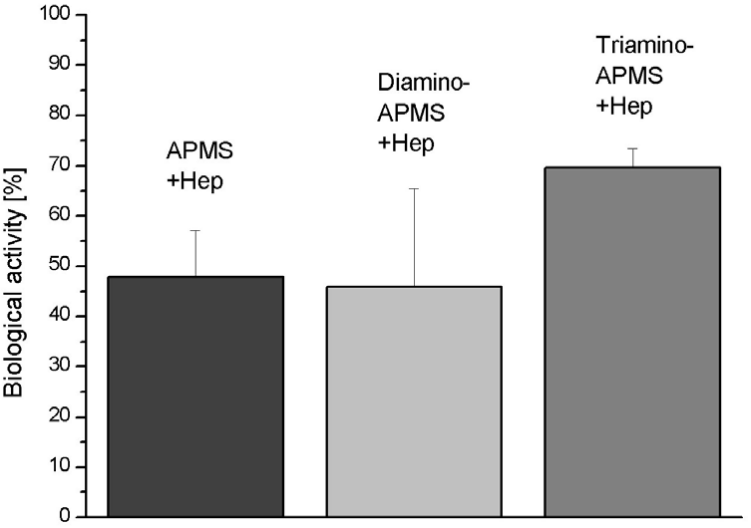
Remaining potencies of heparin immobilised by the spacers APMS, Di- and Triamino-APMS. Each data point is the mean of three samples and error bars are standard deviations.

**Figure 4:**
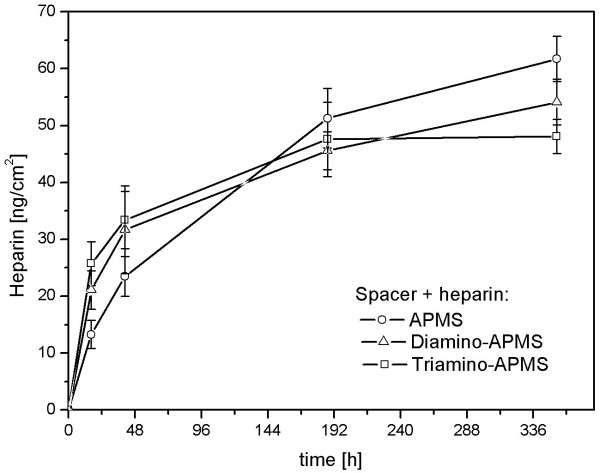
Hydrolysis of heparin from the spacer molecules APMS, Di- and Triamino-APMS in aqueous medium. Each data point is the mean of three samples and error bars are standard deviations.

The potency of the immobilised heparin was measured photometrically by dint of the chromogenic substrate Chromozym TH at λ = 405 nm. Comparing the potencies of the covalently attached heparin on Di- and Triamino-APMS with APMS, it becomes obviously that longer spacer molecules tend to result in higher biological activity of heparin (Figure 3). The influence of the secondary amino groups on the immobilisation of heparin was investigated by measuring the hydrolysis rate of the drug under in vitro simulated physiological conditions (37°C, PBS, 70 shakes per minute). Figure 4 shows that spacers like Triamino-APMS with many secondary amino groups has the highest hydrolysis rate of the drug during the first 100 h, compared to Diamino-APMS and APMS.

**Figure 5:**
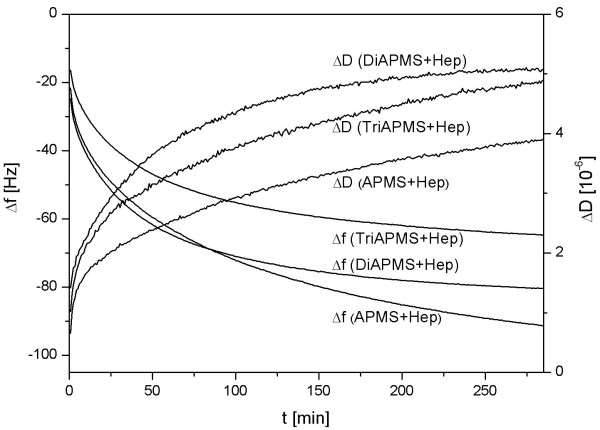
Δf (decreasing curves) and ΔD (increasing curves) of the adsorption of fibrinogen on surfaces functionalised with APMS/Di/Tri(amino-)APMS and Hep(arin) in real-time.

Figure 5 shows changes of the frequency Δf and the dissipation ΔD for the various surfaces during QCM-D measurement. The addition of fibrinogen dissolved in PBS buffer occurred after 5 min and resulted in a decrease of Δf and an increase of ΔD. Since Δf correlates with a mass increase of the crystal due the adsorbed protein layer and ΔD depends on the viscoelastic properties of the layer, the results demonstrate that the amount of adsorbed fibrinogen varied in the range APMS > Diamino-APMS > Triamino-APMS for the differently heparinised surfaces. The amount of adsorbed protein was calculated to be 539 ng/cm2 (APMS), 476 ng/cm2 (Diamino-APMS) and 382 ng/cm2 (Triamino-APMS) after 5 h adsorption time using the Sauerbrey equation. The adsorbed protein layers showed different viscoelastic properties as seen by the dissipation curves, the layer viscosity increased in the range APMS < Triamino-APMS < Diamino-APMS.

## Discussion

The covalent coupling of heparin to titanium dioxide was realised in this study by spacers with different chain lengths like APMS, Di- and Triamino-APMS (Figure 1). The hypothesis was, that the use of longer spacer chains should result in a higher mobility of immobilised heparin and hence a higher biological potency of the drug. Coupling of heparin to the spacer modified surfaces was performed following activation of carboxylic groups with EDC and NHS, which then react with primary amino groups of the spacer molecules (Figure 1a), whereas the secondary amino groups (R-*N*H-R^'^) are not able to form amide bonds with carboxyl groups. The successful modification of TiO_2 _surfaces with coupling agent and heparin was demonstrated by measuring zeta-potential changes in aqueous solution (Figure 2) since both the spacer as well as the drug led to a change of the potential due to charged amino and sulphate groups in the modification.

Although this reaction took place for all three coupling agents, the amount of immobilised spacer and drug was significantly different in the order APMS > Diamino-APMS > Triamino-APMS. This phenomenon can be explained, since the larger molecules like Triamino-APMS are sterically more hindered on the titania surface than the smaller ones. Furthermore, a higher number of secondary amino groups result in a stronger intermolecular electrostatic repulsion. Both effects lead to less pronounced deposition of spacer molecules on the surface. Comparing the values of Table 1 with those of the literature, where a monolayer of NH_2 _groups is described with 2–3 NH_2_/nm^2^, the here accomplished aminations are in the range of multilayers [14]. Furthermore, the number of amino groups on Ti discs was multiple higher than those on the powders (Table 1). However, calculating the surface area of the discs, ideally plane surfaces were supposed, whereas the high micro roughness of the real surface and hence a higher actual surface area was not considered.

A similar behaviour was found for the amount of immobilised heparin which subsequently decreased with increasing the length of the spacer (Table 2). This correlates with the highest density of surface bound primary amino groups of APMS that can form bonds with carboxylic groups of the heparin. If the amount of attached heparin is compared with literature values for heparin modified polymers [15-17] (Table 3), the here obtained values are much lower. This is probably due to the fact, that polymers can bind heparin over their whole volume what can be considered as a heparinisation in the range of many multilayers. Although the amount of immobilised heparin was lower for Triamino-APMS, this coupling agent showed the highest biological potency of the covalently attached drug (Figure 3), which confirmed our hypothesis that longer spacer chains will maintain the biological activity of the material due to a higher flexibility of the immobilised heparin.

**Table 3:** Amount of immobilised heparin on different polymer substrates *(dependent on PEO spacer length)

Substrate	Polyurethane-CO_2_H	Polyurethane-NH_2_	Polyurethane-PEO	Polyethylene
Heparin [μg/cm^2^]	1.40 ± 0.08	2.00 ± 0.13	0.24 ± 0.04–0.47 ± 0.08*	4.4 ± 0.1–4.7 ± 0.1

The influence of the secondary amino groups on the stability of heparin immobilisation was investigated by measuring the hydrolysis rate of the drug under in vitro simulated physiological conditions (37°C, PBS, 70 shakes per minute) (Figure 4). In an aqueous medium, these secondary amino groups are protonated to R_2_NH_2 _^+^, which makes them able to interact with negative sulphate, carboxylate and hydroxyl groups of the heparin via electrostatic interactions. This ionic bonding is weaker than the covalent one and should have only an additional effect. It was shown that spacers like Triamino-APMS with many secondary amino groups had the highest hydrolysis rate of the drug during the first 100 h, compared to Diamino-APMS and APMS. This effect is up to heparin molecules that are only bound electrostatically, which makes the hydrolysis easier and faster. After this period, the hydrolysis profile inverses and the drug hydrolysed slower from Triamino-APMS than from the other ones. The reason probably is, that the remaining heparin molecules are both covalently and ionically bound on the Triamino-APMS compared to pure APMS that can only bind the drug covalently.

The primary reaction after incorporating an implant into the body is the formation of a biofilm composed of adsorbed matrix proteins. The quantitative composition of this protein film and conformational changes during adsorption are thought to be the key factor controlling the attachment of cells and the biological response to the implant material [18]. In case of materials in contact with blood, it is also known that the adsorption of various proteins combined with structural changes of the protein layer, e.g. fibrinogen can initiate blood clothing. The adsorption of fibrinogen to heparinised TiO_2 _surfaces was measured in this study using the QCM-D technique. The QCM-D method offers two advantages, firstly, the adsorption can be followed in real-time due to the mass increase of the quartz crystal and secondly, it is possible to analyse conformational changes due to the viscoelastic properties of the adsorbed protein layer. Therefore, the gold surfaces of the quartz crystals were modified in a first step with a thin (approx. 100 nm) TiO_2 _layer using the PVD technique which were then coated with the coupling agents APMS/Di-/Triamino-APMS and heparin similar to the methods described for titanium substrates. The concentration of the extracellular plasma protein fibrinogen (in PBS, pH = 7.4) used was 50.0 μg/mL. Although this was much lower compared to that of human blood (3 mg/mL), this lower concentration allows single fibrinogen molecules to interact with the surface, before the surface area around the protein is blocked by newly adsorbed fibrinogen molecules. The resulting two effects are firstly a tight bound layer of fibrinogen which contains less water, whereby the effect of water in the protein layer during the QCM-D measurment is reduced. Secondly, lower protein concentrations (< 50 μg/ml) advance surface effects on the protein adsorption and thus the QCM-D measurements provide more information regarding differently functionalised surfaces for protein adsorption [13].

**Figure 6:**
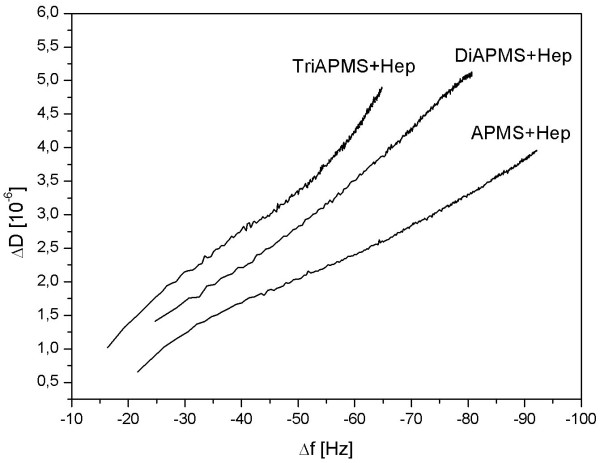
Correlation between ΔD and Δf of the different surfaces from Figure 5. The ΔD/Δf values can be taken to compare the viscosity of protein layers adsorbed onto different surfaces because of elimination of the factor time.

Figure 6 shows a correlation between ΔD and Δf for the differently heparinised surfaces which is helpful for analysing and comparing the viscoelastic properties of protein films on different surfaces since Δf and ΔD did not have the same time dependency such that time as parameter can be eliminated due to this approach. In various experiments it was shown that a low ΔD/Δf is an indication for rigid adsorbed films [19]. The results demonstrate that the ΔD/Δf values for surfaces modified with APMS and heparin nearly built up a line with only a small increase at higher frequencies. In contrast, the ratios of the heparinised surfaces by means of Diamino-APMS and Triamino-APMS are generally higher. This behaviour indicates structural changes within the adsorbed protein layer [20].

Lower ΔD/Δf ratios for the differently heparinised surfaces indicate a more dense package of the protein layer onto the surfaces which is either due to conformational changes of the adsorbed protein layer (denaturation) and/or a high affinity of the binding sites of the protein to the substrate. Fibrinogen is a long rod-like molecule with three different globular sub-domains (E, D and αC domains). Under physiological conditions the overall charge of fibrinogen is negative. The D and E domains are negatively charged but the αC domains are positively charged. The αC domain acts as a pioneer in the surface binding process, but it does not bind strongly to the surface [21]. It seems certain, that the differences for the ΔD/Δf ratios are partially caused by electrostatic interactions between the domains of fibrinogen with the heparin and respectively the different spacer molecules that are bound onto the substrate.

Indeed, it could be figured out through zeta-potential measurements that surfaces from substrates modified with APMS, Di- and Triamino-APMS and the respective subsequent heparinised surfaces (Figure 2) had positive and accordingly negative charges that were all very similar. But even if these overall charges of the respective functionalised surfaces are all in the same range, it is likely, that the secondary amino groups of the spacer molecules Di- and Triamino-APMS interact with the positively charged αC domains of fibrinogen while adsorbing. On the one hand, this could have an influence on the adsorption kinetics of the protein and explain, why the Δf curves of the haparinised surfaces by means of Di- and Triamino-APMS are similar and arise faster compared with that of APMS. On the other hand, this electrostatic interaction could influence conformational changes in the adsorbed protein layer and therefore be responsible for the highest ΔD/Δf ratio for Triamino-APMS.

It is also likely that sterical reasons are responsible for the different ΔD/Δf ratios from Figure 6. Protein layers could be packed denser on surfaces that were modified with the spacer APMS with the shortest molecule chain rather than on Di- and Triamino-APMS modified surfaces, since APMS molecules underlie a lower sterical hindrance among each other. This effect could result in a subsequent denser functionalisation with heparin which finally allows a stronger interaction of fibrinogen with the drug and therefore a denser package, which results in lower ΔD/Δf ratios compared to the heparinisation with longer spacer molecules like Di- and Triamino-APMS. Since a densification of protein films is normally accompanied by conformational changes, it is likely that these changes are less pronounced on the Triamino-APMS surfaces compared to the shorter coupling agents.

## Conclusion

In this comparative study, the heparinisation of titanium dioxide substrates by means of the spacer molecules APMS, Di- and Triamino-APMS was investigated with regard to the biological activity of the immobilised drug and the adsorption of the extracellular plasma protein fibrinogen by means of the QCM-D technique to obtain a first impression of the biocompatibility for a possible in vivo application. The remaining activity of heparin was found to be highest for the covalent attachment with Triamino-APMS as coupling agent due to the long chain of this spacer molecule and therefore the highest mobility of the drug, which allows a better interaction of heparin with ATIII for inhibition of blood clotting. Furthermore, the adsorption of fibrinogen on the differently heparinised surfaces in real time demonstrated that with longer spacer chains and accordingly increasing number of secondary amino groups the ΔD/Δf ratios become higher, which is also associated with a less pronounced denaturation of the protein and thus possibly better biocompatible properties of the substrates in contact with a biosystem.

## Competing interests

The author(s) declare that they have no competing interests.

## Authors' contributions

DT took part in conceiving of the study, carried out the experimental work and drafted parts of the manuscript. RT participated in the design of the study. UG took part in conceiving of the study and drafted parts of the manuscript. All authors read and approved the final manuscript.
